# Oseltamivir-Resistant Pandemic A/H1N1 Virus Is as Virulent as Its Wild-Type Counterpart in Mice and Ferrets

**DOI:** 10.1371/journal.ppat.1001015

**Published:** 2010-07-22

**Authors:** Marie-Ève Hamelin, Mariana Baz, Yacine Abed, Christian Couture, Philippe Joubert, Édith Beaulieu, Nathalie Bellerose, Martin Plante, Corey Mallett, Gregg Schumer, Gary P. Kobinger, Guy Boivin

**Affiliations:** 1 CHUQ-CHUL Research Center in Infectious Diseases and Laval University, Québec City, Québec, Canada; 2 Institut Universitaire de Cardiologie et de Pneumologie de Québec, Québec City, Québec, Canada; 3 GlaxoSmithKline Biologicals, Laval, Québec, Canada; 4 National Microbiology Laboratory, Winnipeg, Manitoba, Canada; National Institutes of Health, United States of America

## Abstract

The neuraminidase inhibitor oseltamivir is currently used for treatment of patients infected with the pandemic A/H1N1 (pH1N1) influenza virus, although drug-resistant mutants can emerge rapidly and possibly be transmitted. We describe the characteristics of a pair of oseltamivir-resistant and oseltamivir-susceptible pH1N1 clinical isolates that differed by a single change (H274Y) in the neuraminidase protein. Viral fitness of pH1N1 isolates was assessed *in vitro* by determining replication kinetics in MDCK α2,6 cells and *in vivo* by performing experimental infections of BALB/c mice and ferrets. Despite slightly reduced propagation of the mutant isolate *in vitro* during the first 24 h, the wild-type (WT) and mutant resistant viruses induced similar maximum weight loss in mice and ferrets with an identical pyrexic response in ferrets (AUC of 233.9 and 233.2, P = 0.5156). Similarly, comparable titers were obtained for the WT and the mutant strains on days 1, 3, 6 and 9 post-infection in mouse lungs and on days 1–7 in ferret nasal washes. A more important perivascular (day 6) and pleural (days 6 and 12) inflammation was noted in the lungs of mice infected with the H274Y mutant, which correlated with increased pulmonary levels of IL-6 and KC. Such increased levels of IL-6 were also observed in lymph nodes of ferrets infected with the mutant strain. Furthermore, the H274Y mutant strain was transmitted to ferrets. In conclusion, viral fitness of the H274Y pH1N1 isolate is not substantially altered and has the potential to induce severe disease and to disseminate.

## Author Summary

During the 2009 pandemic of the novel A/H1N1 (pH1N1) virus, the World Health Organization recommended oseltamivir as first-line agent for treatment of patients with severe infections leading to hospitalization and for those with underlying diseases predisposing to pulmonary complications. Oseltamivir-resistant isolates started to emerge at the end of June 2009 with now more than 100 strains reported worldwide including a few outbreaks where transmission of resistant viruses may have occurred. We characterized the fitness of a pair of oseltamivir-susceptible and oseltamivir-resistant strains emerging from the same familial cluster and that differed by only a single change (H274Y) in the neuraminidase protein. We found that the drug-resistant (mutant) virus was at least as virulent as the drug-susceptible (wild-type) virus in mice and ferrets. Based on these data, we believe that the H274Y pH1N1 mutant strain has the potential to disseminate in the population and to eventually replace the susceptible strain, a phenomenon that has been already observed with seasonal A/Brisbane/59/2007-like (H1N1) viruses.

## Introduction

The novel influenza A (H1N1) virus was initially detected in Mexico and California in April 2009 and then officially became the first pandemic influenza virus of the 21^st^ century on June 11, 2009 [1], [2]. Most confirmed cases of pandemic A/H1N1 (pH1N1) infection have been characterized so far by self-limited flu-like symptoms and signs although a significant proportion of infected patients also presented with vomiting and diarrhea [2]. A minority of cases, notably those involving pregnant women, have been associated with a more severe clinical outcome leading to intensive care admission and death [3], [4], [5]. Mouse, ferret and non-human primate studies have indicated that pH1N1 isolates replicate more efficiently and produce more severe pathological lesions in the lungs than recent human A/H1N1 viruses [6], [7], [8]. Seroprevalence studies have indicated that children were initially serologically naïve to the novel pH1N1 strain whereas some degree of pre-existing immunity to this virus existed in the elderly population [6], [9], [10].

Antivirals are the cornerstone of treatment for severe influenza cases requiring hospitalization and can also be used as prophylactic agents in high-risk individuals. Early reports demonstrated that pH1N1 strains were resistant to the adamantanes due to a S31N mutation in the M2 gene but remained susceptible to neuraminidase inhibitors (NAIs) such as oseltamivir and zanamivir [6], [11]. However, oseltamivir resistance has been on the rise in recent seasonal influenza A/H1N1 viruses. Indeed, during the 2008–09 influenza season, almost all characterized influenza A/Brisbane/59/2007-like (H1N1) strains from North America and Europe were resistant to oseltamivir due to a H274Y (N2 numbering) mutation in the neuraminidase (NA) gene [12], [13], [14]. The sudden and large dissemination of this mutant A/H1N1 virus occurred in the apparent absence of antiviral pressure suggesting that it had no impairment in viral fitness. This drug resistance mutation has also been reported in some A/H5N1 viruses [15], [16] and, more recently, in several pH1N1 strains recovered from both immunocompromised and immunocompetent subject [17], [18], [19], [20]. We recently reported the emergence of such an oseltamivir-resistant H274Y mutant in a familial cluster of pH1N1 infections [21]. In this outbreak, we identified a drug-susceptible virus recovered before therapy from a 13-year old boy and a drug-resistant virus collected a few days later from his father who was receiving oseltamivir prophylaxis. We now describe the *in vitro* and *in vivo* replicative characteristics of the drug-resistant and wild-type (WT) viruses isolated from this outbreak.

## Results

As shown in Table 1, the pH1N1 isolate from the index case collected before oseltamivir therapy (A/Québec/147023/2009-WT) was susceptible to all NAIs whereas the pH1N1 isolate from the contact case recovered during post-exposure oseltamivir prophylaxis (A/Québec/147365/2009-H274Y) was resistant to oseltamivir and peramivir. Both isolates were susceptible to zanamivir and A-315675 similarly to 20 other pH1N1 isolates collected from untreated subjects in the same period. The pattern of NAI resistance of the pH1N1 H274Y mutant was similar to that of another H274Y mutant from a seasonal A/H1N1 strain (A/Brisbane/59/2007-H274Y). A pH1N1 H274Y recombinant mutant virus generated from an unrelated pH1N1 strain also exhibited high levels of resistance to oseltamivir and peramivir but remained susceptible to zanamivir and A-315675 (Table 1).

**Table 1:** Susceptibility profiles of pH1N1 isolates, A/Brisbane/59/2007-like A/H1N1 isolates of the 2009 season, and recombinant pH1N1 viruses as tested by NA inhibition assays.

	Oseltamivir IC50 in nmol/L ± SD^a^	Zanamivir IC50 in nmol/L ± SD^a^	Peramivir IC50 in nmol/L ± SD^a^	A-315675 IC50 in nmol/L ± SD^a^
**Seasonal A/H1N1 isolates of the 2009 season (n = 33)**	541.9±73.17	0.36±0.2	33.1±8.1	1.71±0.31
**pH1N1 isolates of the 2009 season (n = 20)**	0.43±0.17	0.12±0.04	0.08±0.03	0.17±0.04
**A/Québec/147023/2009-WT (pH1N1)**	0.27±0.01 (1)	0.18±0.01 (1)	0.08±0.01 (1)	0.08±0.004 (1)
**A/Québec/147365/2009-H274Y (pH1N1)**	423.01±20 (1566)	0.12±0.01 (0.6)	11.51±2.7 (144)	0.28±0.07 (3.5)
**A/Brisbane/59/2007-WT (seasonal)**	0.53±0.1 (1)	0.3±0.04 (1)	0.1±0.03 (1)	0.32±0.12 (1)
**A/Brisbane/59/2007-H274Y (seasonal)**	751.12±93 (1417)	0.5±0.2 (1.7)	34.5±9.2 (345)	0.82±0.3 (2.6)
**Recombinant-WT (pH1N1)**	0.46±0.01 (1)	0.15±0.01 (1)	0.09±0.01 (1)	0.2±0.03 (1)
**Recombinant-H274Y (pH1N1)**	451.9±26 (982)	0.14±0.01 (1)	23.63±5.5 (268)	0.28±0.01 (1.4)

a IC50 values were determined in triplicate experiments. Mean IC50 ± standard deviation (SD) are shown. Fold increases in IC50 values compared to the respective WT virus are shown in parentheses.

Abbreviations: pH1N1, pandemic A/H1N1 virus; WT, wild-type; NA, neuraminidase.

Sequence analysis of the original clinical isolates revealed the presence of only one substitution (H274Y; N2 numbering) in the NA gene of the contact case (GenBank accession number FN434454) compared to that of the index case (accession number FN434445). There was no change in the remaining 7 segments between these two strains (accession number FN434440 to FN434447 for the index case virus and FN434448 to FN4456 for the contact case virus). Phylogenetic analysis of the NA and HA genes showed that the two pH1N1 isolates described in this study were closely related to pH1N1 strains identified in North America, Europe and Asia (data not shown). The viral populations in the two clinical isolates were homogenous as 100% (16/16) of clones from the index case had the H274 sequence whereas 100% (16/16) of clones from the contact case harboured the 274Y sequence.

**Figure 1:**
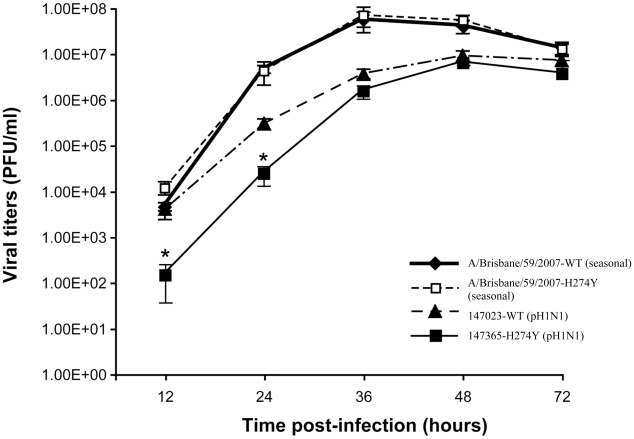
Replicative capacities of wild-type (WT) and H274Y mutant isolates of pH1N1 and seasonal A/H1N1 viruses. Viral titers were determined at the indicated time points from supernatants of ST6Gal I-expressing MDCK cells infected with pH1N1 A/Québec/147023/2009 (WT), pH1N1 A/Québec/147365/2009 (H274Y mutant), A/Brisbane/59/07-like (WT) and A/Brisbane/59/07-like (H274Y mutant) isolates at a multiplicity of infection (MOI) of 0.001. Mean viral titers ± SD from triplicate experiments were determined by using standard plaque assays. ^*^ *P*<0.05 between the WT and H274Y pH1N1 viral titers.

*In vitro* experiments performed in MDCK cells expressing the α2,6 sialic acid receptor indicated that the oseltamivir-resistant pH1N1 isolate replicated less efficiently than the WT pH1N1 during the first 24 h. However, there was no significant difference in viral titers subsequently i.e. from 36 to 72 h (Figure 1). The two pH1N1 isolates produced lower viral titers than seasonal A/H1N1 viruses (A/Brisbane/59/2007) including both a WT and a H274Y mutant at 36 and 48 h. Thus, the H274Y mutation resulted in either no impairment or only initial reduction in replicative capacities when inserted in seasonal and pandemic A/H1N1 backgrounds, respectively. Of note, the two pH1N1 viruses produced less well defined viral plaques on α2,6-transfected MDCK cells compared to seasonal strains (data not shown).

**Figure 2:**
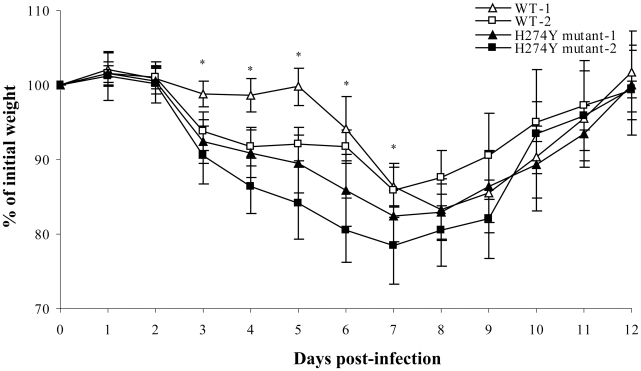
Weight loss of mice infected with wild-type (WT) or H274Y mutant isolates of pH1N1. The weight loss of mice was evaluated in two groups of 15 mice (infected with the WT and H274Y mutant isolate) over a period of 14 days. These experiments were performed twice and reported as #1 and #2 on the graph. * *P*<0.05 between the WT and H274Y pH1N1 weight losses.

**Figure 3:**
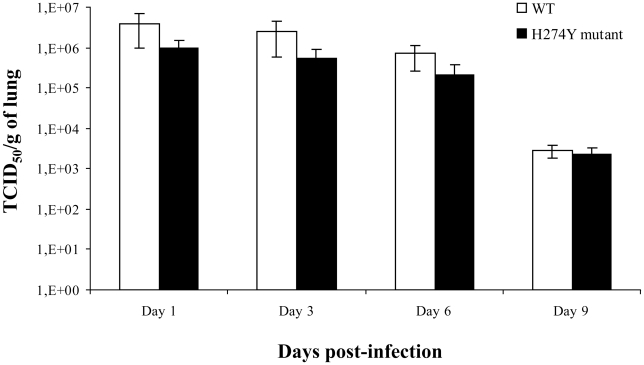
Lung viral titers of mice infected with wild-type (WT) or H274Y mutant isolates of pH1N1. Mice were infected intranasally with 5×10^5^ PFUs of either WT pH1N1 (A/Québec/147023/2009) or oseltamivir-resistant H274Y mutant (A/Québec/147365/2009) isolate. Three to four mice per group were sacrificed on days 1, 3, 6 and 9 for determination of lung viral titers, which were reported as TCID_50_ per gram of lung.

Two separate mouse experiments were conducted to assess weight loss, clinical signs, viral titers (on days 3 and 6 in the first experiment and on days 1, 6 and 9 in the second experiment) and histopathological changes. In the first experiment, the WT and oseltamivir-resistant pH1N1 isolates induced similar maximum weight loss, which peaked on day 8 at 16.3% for both groups (*P* = 0.81) although there was a more pronounced weight loss from days 3 to 7 with the mutant strain (Figure 2). In the second experiment, more weight loss was induced after infection with the H274Y mutant from days 3 to 8. By day 12, all mice from the two experiments had returned to their initial weight with no mortality. Lung viral titers, which were determined on days 1, 3, 6 and 9 post-infection, did not significantly differ between the WT and H274Y mutant viruses when assessed by quantitative viral culture (Figure 3) and real-time RT-PCR (Figure S1). Importantly, there was no unexpected change in the NA sequence of viruses recovered from lungs of euthanized mice.

**Figure 4:**
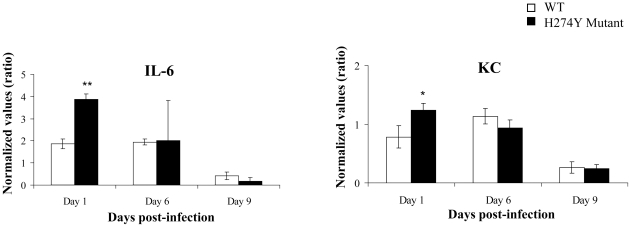
Lung IL-6 and KC expression in mice infected with wild-type (WT) or H274Y mutant isolates of pH1N1. Mice were infected intranasally with 5×10^5^ PFUs of either WT pH1N1 isolate (A/Québec/147023/2009) or oseltamivir-resistant H274Y mutant (A/Québec/147365/2009). Three to four mice per group were respectively sacrificed on days 1, 6 and 9 for determination of lung IL-6 and KC transcripts. ^*^ *P*<0.05, ** *P*<0.0001 between the WT and H274Y cytokine transcript levels.

Transcript levels for various cytokines/chemokines (KC [CXCL1], MCP-1 [CCL2], MIP-1α, IFN-γ, IL-4, IL-5, IL-6 and IL-10) were determined in lungs of infected mice on days 1, 6 and 9 post-infection. All cytokines/chemokines were equally expressed following infection with either of the two pH1N1 isolates (Figure S2), with the exception of increased expression of IL-6 and KC levels on day 1, following infection with the H274Y mutant virus (Figure 4).

**Figure 5:**
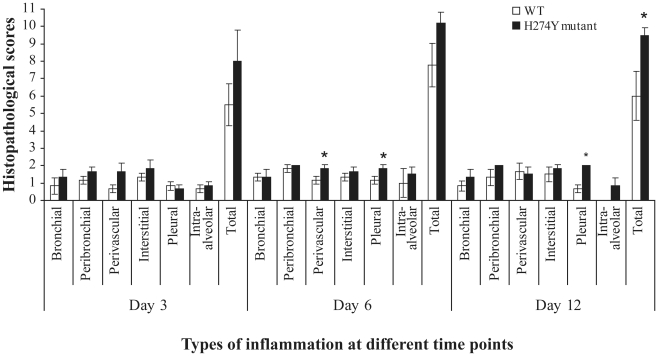
Lung histopathology of mice infected with wild-type (WT) or H274Y mutant isolates of pH1N1. Groups of mice were euthanized on days 3, 6 and 12 post-infection and one pulmonary lobe was removed and fixed with 10% formalin. Thin sections of paraffin-embedded lung tissues were cut and stained with hematoxylin and eosin. The degree of lung inflammation (mean histopathological score) was evaluated for bronchial, peribronchial, perivascular, interstitial, pleural and intra-alveolar inflammation. ^*^ *P*<0.05 between the WT and H274Y lung histopathological scores.

Both pH1N1 isolates induced significant pulmonary inflammation including peribronchial, interstitial, perivascular, alveolar and pleural inflammation that peaked on day 6 post-infection (Figure S3). There was significantly more perivascular (day 6) and pleural (days 6 and 12) inflammation visualized in the lungs of mice infected with the H274Y mutant compared to the WT virus (Figure 5). A mild to moderate vascular congestion was observed in both groups of mice although pulmonary oedema was not noted in any mice. Inflammatory cellular infiltration was characterized by both acute (neutrophilic) and chronic (lymphohistiocytic) infiltrates in all mice. Thus, mouse experiments indicated that the mutant pH1N1 isolate induced more pronounced weight loss than the WT virus which correlated with increased expression of IL-6 and KC and more significant lung inflammation despite similar lung viral titers.

**Figure 6:**
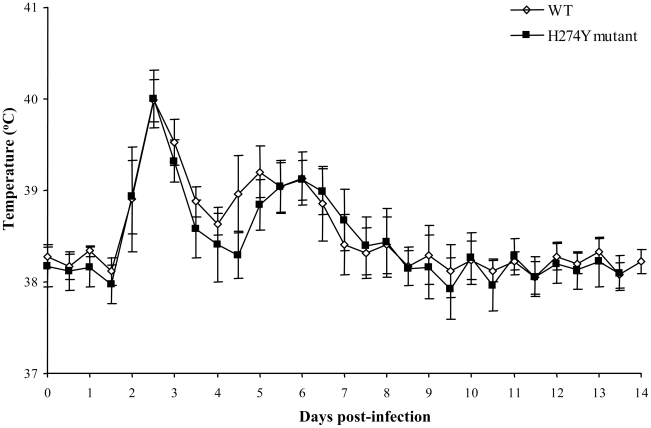
Body temperature of ferrets infected with wild-type (WT) or H274Y mutant isolates of pH1N1. Body temperatures were recorded by implanted thermometers during 14 days post-inoculation in groups of 4–5 ferrets infected with WT (A/Québec/147023/2009) or oseltamivir-resistant H274Y mutant (A/Québec/147365/2009) pH1N1 isolates.

Intranasal inoculation of ferrets with the WT and H274Y mutant pH1N1 isolates resulted in a strong anti-A/California/07/2009 serum antibody response on day 14 (hemagglutination inhibition reciprocal geometric mean titers went from <20 to 4208 and from <20 to 3135, respectively). Notably, all ferrets had preexisting HI antibodies against seasonal A/H1N1 (A/Brisbane/59/07) but titers were similar in the two groups of ferrets pre- and post-infection (Table S1). A pyrexic response was seen between days 2 and 8 post-inoculation (Figure 6). Interestingly, temperature curves were biphasic with a major peak on days 2–3 and another lower peak on days 5–6 in both groups of ferrets. The area under the curve (AUC) of temperatures over the course of the 14-day experiment was similar for both groups of ferrets i.e. 233.9±0.5787 for the WT and 233.2±0.8669 for the H274Y mutant (*P* = 0.5156). Also, the mean percentage of body weight loss over time was not significantly different in animals infected with the WT or the H274Y mutant virus (Figure S4). The maximum weight loss (day 7 and day 3) was 7.54% and 4.15% for the WT and H274Y mutant viruses, respectively (*P* = 0.0515). By the end of the 14-day observation period, the ferrets had returned to their initial weight with no mortality.

**Figure 7:**
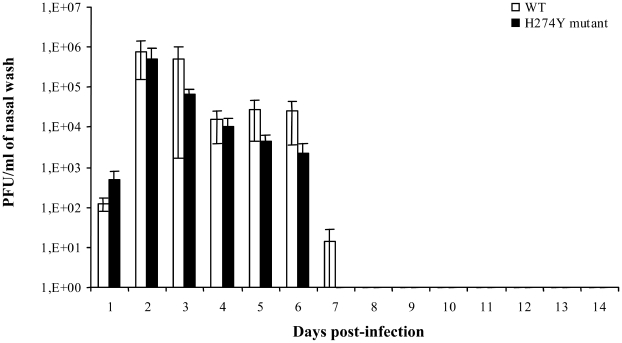
Nasal wash viral titers of ferrets infected with wild-type (WT) or H274Y mutant isolates of pH1N1. Viral titers were determined daily over 14 days post-inoculation from nasal washes of ferrets infected with WT (A/Québec/147023/2009) or oseltamivir-resistant H274Y mutant (A/Québec/147365/2009) pH1N1 isolates. Mean viral titers ± SD from triplicate experiments were determined by using standard plaque assays.

Viruses could be recovered from nasal wash of ferrets up to 7 days post-infection with a peak on day 2 post-infection (Figure 7). Viral titers did not significantly vary at any time points when comparing the two groups of ferrets. Increased levels of IL-6, IL-12 and IFN-γ mRNA were observed in retropharyngeal lymph nodes of ferrets infected with the H274Y mutant compared to the WT on day 14 with ratios of 1.174, 1.38 and 1.183, respectively (not shown). Expression of IL-2 was decreased in ferrets infected with the mutant virus compared to the WT with a ratio of 0.8. Thus, ferret experiments showed no significant differences in clinical parameters (temperature and weight) and viral titers in the upper respiratory tract for the WT and mutant pH1N1 isolates. However, some cytokines (IL-6, IL-12 and IFN-γ) were specifically upregulated in lymph nodes of ferrets infected with the H274Y mutant.

A limited transmission study was conducted in ferrets and it demonstrated that the H274Y mutant strain was transmitted from ferrets experimentally infected intranasally to ferrets placed in the same cage 24 h after infection. All contact ferrets seroconverted for A/California/07/2009 when tested 14 days after contact (hemagglutination inhibition reciprocal geometric mean titers went from <20 to 993). All contact ferrets also shed virus and had a mean peak viral titer of 8.32×10^4^ PFU/ml in their nasal washes.

## Discussion

Oseltamivir, the most frequently used NAI, is recommended for treatment of patients with severe pH1N1 infections leading to hospitalization or those with underlying diseases which place them at risk of complications. We have recently described the rapid emergence of oseltamivir resistance in a family cluster of pH1N1 infection due to the H274Y NA mutation [21], and now report on the viral fitness of this mutant *in vitro* and *in vivo*. Despite slightly reduced propagation of the mutant isolate *in vitro* during the first 24 h of infection compared to the original WT virus, both isolates replicated as efficiently in the lower respiratory tract of mice and in the upper respiratory tract of ferrets inducing similar maximum weight loss and pyrexic response between days 2 and 8 post-infection. Interestingly, the H274Y NA mutant induced a slightly more pronounced weight loss than the WT virus in mice, and this observation correlated with increased production of IL-6 and KC and a more important pulmonary inflammation involving the perivascular (day 6) and pleural (days 6 and 12) compartments.

Previous studies have demonstrated that oseltamivir resistance results from subtype-specific NA mutations [15]. In influenza viruses of the N1 subtype, including seasonal A/H1N1 viruses and avian A/H5N1 strains, oseltamivir resistance is mainly conferred by the H274Y (N2 numbering) mutation [16], [22], [23]. This mutation has also been recently detected in pH1N1 viruses [17], [18], [19], [20], [21]. The larger tyrosine residue at codon 274 prevents the re-orientation of glutamic acid at position 276 within the catalytic site, which is required to accommodate the bulky side chain of oseltamivir but not zanamivir [24]. In agreement with previously-described seasonal A/H1N1 viruses containing the H274Y NA mutation, our oseltamivir-resistant pH1N1 mutant showed cross-resistance to peramivir, a parenteral NAI that is in phase 3 clinical trials [15], [25] and also readily available through an emergency access program [26]. On the other hand, our phenotypic findings demonstrate an interesting potential for inhaled zanamivir and the investigational orally-available A-322278 NAI compound (the prodrug of A-315675) [27], as alternative agents for treatment of oseltamivir-resistant pH1N1 infections.

The impact of mutations conferring NAI resistance on viral fitness and transmissibility may vary depending on the genetic background of influenza viruses. In the N1 subtype, the H274Y mutation has been initially reported as impairing the viral fitness of older seasonal strains such as A/New Caledonia/20/99-like and A/Texas/36/91 when evaluated in the ferret model [28], [29]. However, transmission of the H274Y mutant strain was documented in ferrets [28]. More recently, our group showed that the same mutation had a different effect, i.e. it was associated with conserved viral fitness in the seasonal A/Brisbane/59/2007 (H1N1) background when assessed both *in vitro* and in ferrets [30]. In the present study, the replication of the pH1N1 H274Y mutant was initially impaired *in vitro* compared to the WT virus but viral titers were virtually identical on days 2 and 3 post-infection. The two pH1N1 isolates replicated less efficiently (lower titers and reduced plaque formation) than the recent WT and H274Y mutant A/Brisbane/59/2007 strains in ST6Gal I-expressing MDCK cells, which may indicate a greater affinity of the seasonal strain for α2,6 sialic acid receptors.

Different groups have reported the use of exprimental animal models for studying WT pH1N1 infection. For instance, Itoh et al. [6] and Maines et al. [7] observed significant weight loss in BALB/c mice associated with efficient viral replication in lungs when an intranasal inoculum ≥10^4^ plaque forming units (PFUs) was used. Some mice even died from a viral challenge consisting of 10^6^ PFUs [6]. Those results indicate that pH1N1 strains can replicate efficiently in mice in contrast to most seasonal A/H1N1 strains. The selective replication of pH1N1 virus in the BALB/c mouse model without prior animal adaptation is reminiscent of features described for highly pathogenic A/H5N1 viruses and the 1918 Spanish flu virus although they are generally less severe with pH1N1 [31], [32]. In agreement with these reports, we observed that pH1N1 can efficiently infect BALB/c mice inducing weight loss, high viral lung titers and also significant pulmonary histopathological changes. Moreover, we now report that the H274Y pH1N1 mutant virus is clearly as fit as the WT virus in this animal model. In fact, more weight loss was induced by the H274Y mutant compared to the WT virus during the first 7–8 days post-infection although all mice eventually returned to their initial weight. On the other hand, we found no significant differences in lung viral titers between the two groups of mice when assessed on days 1, 3, 6 and 9. The more prounounced weight loss observed with the mutant strain compared to the WT virus was confirmed in two separate experiments using similar viral inoculum (as confirmed by back titration) and unaltered viral genomes (as confirmed by sequencing the virus from lungs of euthanized mice). These important clinical signs correlated with slightly more severe histopathological changes observed in lungs of mutant-infected mice in particular on days 6 and 12 post-infection and more specifically in the perivascular and pleural compartments. Altogether, those results suggest that the H274Y pH1N1 mutant isolate stimulated a more important inflammatory response in mice compared to WT virus, which could be due to rapid induction of IL-6 and KC in the former. It has been reported for different influenza viruses that the early secretion of pro-inflammatory cytokines was associated with the development of pulmonary inflammation at a later stage [33] as shown here for pH1N1. Interestingly, the H274Y mutant also induced preferential expression of IL-6, IL-12 and IFN-γ in the retropharyngeal lymph nodes of ferrets compared to the WT virus. In humans, IL-6 has been shown to be the first cytokine to appear in nasal wash of infected individuals and the one likely responsible for much of the clinical symptoms [34], [35], [36]. Additional studies are required to assess the mechanism leading to increased IL-6 levels in animals infected with the H274Y NA mutant.

As shown in some but not all studies [6], [7], [8], our two pH1N1 isolates induced a strong pyrexic response (increased in temperature of 2°C) and slight weight loss (3–7%) in ferrets. Interestingly, we noticed a biphasic temperature curve for both groups of ferrets. The first and major febrile peak correlated with maximum viral titers and the second minor increase in temperature seen on day 6 might be due to cytokine release. Similar biphasic temperature curves were also seen in ferrets infected with WT A/H5N1 viruses (data not shown). Previous investigators have shown that both pH1N1 and seasonal A/H1N1 strains replicate to similar levels in the upper respiratory tract of ferrets but only the former could replicate to high levels in the lungs [6], [7], [8]. As for the comparison of the WT and mutant pH1N1 isolates, we found no significant difference in nasal wash viral titers of ferrets as determined at several time points but did not assess lung viral titers.

Among the strengths of our study is the use of two clinical isolates from the same familial cluster that differed by a single a.a. and that were only passaged twice before animal studies. Also, the use of two different animal models to characterize the virulence of these strains and the relatively similar results observed in both of them reinforce our conclusions. A limitation of our study is the incomplete assessment of the transmissibility of our pH1N1 strains. Some groups have shown that the WT pH1N1 strain can be transmitted efficiently via aerosol or respiratory droplets [6], [8]. We report here that the H274Y mutant could be transmitted by contact to uninfected ferrets but did not compare the efficiency of transmission with the WT strain and neither evaluated aerosol transmission. A confounder is the seropositive status of our ferrets for the seasonal A/Brisbane/59/2007 (H1N1) strain before challenge with pH1N1 viruses. In guinea pigs, preexisting immunity to recent seasonal A/H1N1 viruses reduced viral load and transmission of pH1N1 [37] whereas a ferret study with the seasonal A/H1N1 vaccine showed little protection from challenge with pH1N1 [38]. Although preexisting antibody levels against an heterologous strain could have an effect on pathogenicity and transmission of pH1N1, the geometrical mean antibody titers were similar for our two groups of ferrets, which should not change our conclusion about the relative pathogenicity of the H274Y mutant compared to the WT strain. Furthermore, we found similar results with the two strains in non-immune mice. Finally, our animal results may not be completely relevant to humans due to differences in distribution of HA cell receptors.

In summary, although some slight differences were observed in the two animal models, we can conclude that the H274Y pH1N1 mutant seems as virulent as the WT isolate with no obvious impairment in viral fitness. Although reports of limited person-to-person transmission in several epidemiological settings have been observed [39], currently no evidence of widespread dissemination of oseltamivir-resistant pH1N1 has been reported indicating the continued value of this drug for treatment of severe cases. Other H274Y pH1N1 mutants (with different genetic backgrounds) should be studied in terms of virulence and efficiency of transmission to confirm our conclusions. In the meantime, careful monitoring of the H274Y mutation during pH1N1 outbreaks is mandatory to rapidly identify transmission events that could lead to large-scale dissemination of an oseltamivir-resistant pH1N1 strain.

## Materials and Methods

### Viruses

The two pH1N1 viruses that differed by only a single mutation in the NA gene were recovered from a family cluster of infection and passaged twice in ST6Gal I-expressing MDCK cells before testing. The two seasonal A/H1N1 viruses were collected in 2007 and passaged 3 times before testing.

### Drug susceptibility assays

Susceptibility profiles of pandemic and seasonal influenza A/H1N1 strains as well as recombinant pH1N1 viruses against oseltamivir carboxylate (Hoffmann La Roche, Basel, Switzerland), zanamivir (GlaxoSmithKline, Stevenage, UK), peramivir (BioCryst, Birmingham, AL) and A-315675 (Abbott Laboratories, North Chicago, IL) were determined by NA inhibition assays using methylumbelliferyl-N-acetyl neuraminic acid (MUNANA, Sigma, St. Louis, MO) as a fluorescent substrate [40].

### Generation of recombinant pH1N1 viruses

The eight segments of pandemic influenza A/H1N1 virus (A/Quebec/141447/09) were amplified by RT-PCR and inserted into the recently-described bidirectional pLLB-A/G expression/translation plasmids by recombination in *E. coli* [41]. The pLLBA plasmid containing the NA segment was used in PCR-mediated site-directed mutagenesis kit (Stratagene, La Jolla, CA) for the introduction of the H274Y mutation. Plasmids were then used to cotransfect 293T cells for the rescue of recombinant viruses as previously described [42].

### Viral genome sequencing and evaluation of viral quasispecies

RNA was isolated directly from human nasopharyngeal aspirates or mouse lungs by using the QIAamp Viral RNA kit (Qiagen, Mississauga, ON, Canada). Complementary DNA (cDNA) was synthesized by using 500 ng of the influenza specific Uni12 primer [43] and the SuperScript II reverse transcriptase (GIBCO-BRL, Burlington, ON, Canada). Viral cDNA was used to amplify the eight influenza viral segments by PCR using the *Pfu Turbo Polymerase* (Stratagene, La Jolla, CA) and primers specific for each influenza gene [43]. PCR products were gel-purified and sequenced using an automated DNA sequencer (ABI Prism 377 DNA sequencer, Applied Biosystems, Foster City, CA). For evaluation of viral quasispecies, cDNAs of the NA gene from the original clinical samples were cloned into the pJET vector (Fermentas, Burlington, ON). Sixteen clones were sequenced to establish the ratios of WT and mutant populations within each clinical strain.

### *In vitro* replication experiment

ST6Gal I-expressing MDCK cells (kindly provided by Dr. Y. Kawaoka, University of Wisconsin, WI) [44] were infected at a multiplicity of infection (MOI) of 0.001 with pandemic or seasonal A/H1N1 viruses (A/Brisbane/59/2007) containing or not the H274Y NA mutation. Supernatants were serially collected post-infection and assayed for numbers of PFUs using standard plaque assays.

### Mouse studies

Six to eight week old female BALB/c mice (Charles River, ON, Canada) were used to evaluate weight loss as well as lung viral titers, cytokines transcript levels and histopathological changes following pH1N1 infection. Anesthetized mice were challenged by intranasal inoculation of 5×10^5^ PFUs in 50 µl virus diluent (Minimal Essential Media (MEM), 0.3% Bovine Serum Albumin (BSA), penicillin/streptomycin) of the WT or H274Y mutant influenza virus isolate. After challenge, animals were weighed daily for 12 days and monitored for clinical signs. On days 1, 3, 6 and 9 post-infection (days varied according to the experiment), 3 mice per group were sacrificed and lung tissue was placed into RNAlater (Qiagen) for RNA preservation and subsequent RNA extraction. Additional samples of fresh tissues were immediately frozen for viral isolation. All procedures were approved by the Institutional Animal Care Committee at the National Microbiology Laboratory (NML) of the Public Health Agency of Canada (PHAC) according to the guidelines of the Canadian Council on Animal Care. All infectious work was performed in biocontainment level 4 at the NML.

Lung tissues were harvested during necropsies and homogenized in MEM/BSA using a bead mill homogenizer (Tissue Lyser, Qiagen). Debris was pelleted by centrifugation (2,000 g, 5 min) and 10-fold serial dilutions of supernatant were plated on MDCK cells with six replicates per dilution. At 72–96 h post-infection, the plates were scored for cytopathic effects (CPE) and the TCID_50_ virus titers were calculated using the method of Reed and Muench [45]. Tissues preserved in RNAlater were homogenized using a bead mill homogenizer and RNA was isolated using the RNeasy Mini Kit (Qiagen). Pandemic H1N1 copy numbers were determined by Q-RT-PCR using the LightCycler 480 RNA Master Hydrolysis Probes (Roche Diagnostics, Laval, QC) assay targeting the hemagglutinin gene (nt position 714–815, GenBank number GQ160606). Reaction conditions were the following: 63°C – 3 min, 95°C – 30 s and cycling of 95°C – 15 s, 60°C – 30 s for 45 cycles using a Lightcycler 480 (Roche). The lower detection limit for this pH1N1 assay is 0.1 PFU/ml. The primer/probe sequences are as follow: HAForward– GGATCAAGAAGGGAGAATGAACTATT; HAReverse – AATGCATATCTCGGTACCACTAGATTT and HAProbe (TET) – CCGGGAGACAAAA-TAACATTCGAAGCAAC.

For measuring cytokines expression on days 1, 6 and 9, extracted RNA was first analyzed with the RNA 6000 Nano LabChip and Bioanalyser (Agilent, Switzerland). cDNA was prepared using RNA of standardized quality (RIN>8) and quantity (3.68 µg of total RNA), the Superscript II RNase H (Invitrogen, Burlington, ON, Canada) and 250 ng of random primer hexamers (Invitrogen). Equal amounts of cDNA were run in triplicate and amplified/detected using the Amplifluor UniPrimer system (Applied Biosystem, Foster City, CA) in which the forward or the reverse primers are tailed with the Z sequence 5′-ACTGAACCTGACCGTACA. The results were normalized based on amplification of an internal gene (18S ribosome) and amounts of target gene were calculated according to a standard curve. The primer sequences for the cytokines/chemokines IL-4, IL-5, IL-6, IL-10, KC, MCP-1, MIP-1α and IFN-γ are available upon request [46].

For lung histopathological studies, one pulmonary lobe was removed at serial times and fixed with 10% buffered formalin. Tissues were embedded in paraffin, sectioned in slices of 4 µm and stained with hematoxylin-eosin. The histopathological scores (HPS) were determined by two independent pathologists with experience in pulmonary pathology who were unaware of the infection status of the animals. A semi-quantitative scale was used to score bronchial/endobronchial, peribronchial, perivascular, interstitial, pleural and intra-alveolar inflammation [47]. Capillary vascular congestion and pulmonary edema were also evaluated using a semi-quantitative scale and the inflammatory cellular infiltrate was characterized to determine if the inflammation was acute (neutrophilic) or chronic (lymphohistiocytic).

### Ferret virulence studies

Groups of five male ferrets (900–1500 g) (Triple F Farms, Sayre, PA) were lightly anesthetized by isoflurane and received by intranasal instillation 250 µl (125 µl/nare) of PBS containing 4.5log TCID_50_/ml of pH1N1 viruses with or without the H274Y NA mutation. Telemetric transmitters (DST micro-T, Star-Oddi, Iceland) were subcutaneously implanted and temperature profiles of ferrets were recorded every 15 min starting 2 days prior and up to 14 days post-inoculation. Ferrets were weighed daily and nasal wash samples were collected from animals on a daily basis during 14 days post-inoculation by instillation of 5 ml of PBS into the external nares of the animals. The work was performed in biocontainment level 2+ according to procedures approved by the Institutional Animal Care Committee of Armand Frapier Institute.

Virus titers were determined by standard plaque assays using ST6Gal I-expressing MDCK cells. In addition, serum was also collected from each ferret before intranasal infection and on day 14 post-infection to evaluate specific antibody levels against the pH1N1 A/California/07/2009 and the seasonal A/Brisbane/59/2007 strains using standard hemagglutination inhibition assays.

For transcripts analysis of IL-2, IL-6, IL-12 and IFN-γ on day 14 post-infection, RNA was isolated from retropharyngeal lymph nodes using the RNAqueous Micro (Ambion, Streetsville, Ontario) and cDNA was generated with the Transcriptor First Strand cDNA Synthesis Kit (Roche). Amplification of cytokine cDNA was performed as previously described [48]. The results were normalized based on amplification of an internal gene (GAPDH).

### Ferret transmission studies

Five male ferrets (900–1500 g) (Triple F Farms, Sayre, PA) were lightly anesthetized by isoflurane and received by intranasal instillation 250 µl (125 µl/nare) of PBS containing 4.5log TCID_50_/ml of pH1N1 virus with the H274Y NA mutation. Each ferret was placed individually in a cage. Approximately 24 h following viral infection, inoculated-contact animal pairs were established by placing a naïve ferret into each cage allowing the exchange of respiratory droplets by direct contact. Inoculated and contact animals were monitored for clinical signs and nasal wash samples were collected for viral titers every day over a 14-day period. Serum was also collected from each ferret before intranasal infection and on day 14 post-infection to evaluate specific antibody levels against the pH1N1 A/California/07/2009 virus using standard hemagglutination inhibition assays. All ferrets were seronegative for pH1N1 A/California/07/2009 before intranasal infection.

### Statistical analyses

Paired and unpaired *t* test analyses were done to compare the mutant and WT virus characteristics during *in vitro* and *in vivo* studies, respectively.

## Supporting Information

Figure S1 Lung viral titers of mice infected with wild-type (WT) or H274Y mutant isolates of pH1N1.
(0.23 MB TIF)

Figure S2 Lung cytokines/chemokines expression in mice infected with wild-type (WT) or H274Y mutant isolates of pH1N1.
(0.41 MB TIF)

Figure S3 Lung histopathology of mice infected with wild-type (WT) or H274Y mutant isolates of pH1N1.
(5.84 MB TIF)

Figure S4 Weight loss of ferrets infected with wild-type (WT) or H274Y mutant isolates of pH1N1.
(0.15 MB TIF)

Table S1 Hemagglutination inhibition titers for pH1N1 (A/California/7/09) and seasonal A/H1N1 (A/Brisbane/59/07) in ferrets infected with wild-type and H274Y mutant pH1N1 viruses.
(0.04 MB DOC)

## References

[1] (2009). Outbreak of swine-origin influenza A (H1N1) virus infection - Mexico, March-April 2009.. MMWR Morb Mortal Wkly Rep.

[2] Dawood FS, Jain S, Finelli L, Shaw MW, Lindstrom S (2009). Emergence of a novel swine-origin influenza A (H1N1) virus in humans.. N Engl J Med.

[3] (2009). Hospitalized patients with novel influenza A (H1N1) virus infection - California, April–May, 2009.. MMWR Morb Mortal Wkly Rep.

[4] Davies A, Jones D, Bailey M, Beca J, Bellomo R (2009). Extracorporeal membrane oxygenation for 2009 influenza A(H1N1) acute respiratory distress syndrome.. JAMA.

[5] Jamieson DJ, Honein MA, Rasmussen SA, Williams JL, Swerdlow DL (2009). H1N1 2009 influenza virus infection during pregnancy in the USA.. Lancet.

[6] Itoh Y, Shinya K, Kiso M, Watanabe T, Sakoda Y (2009). In vitro and in vivo characterization of new swine-origin H1N1 influenza viruses.. Nature.

[7] Maines TR, Jayaraman A, Belser JA, Wadford DA, Pappas C (2009). Transmission and pathogenesis of swine-origin 2009 A(H1N1) influenza viruses in ferrets and mice.. Science.

[8] Munster VJ, de Wit E, van den Brand JM, Herfst S, Schrauwen EJ (2009). Pathogenesis and transmission of swine-origin 2009 A(H1N1) influenza virus in ferrets.. Science.

[9] (2009). Serum cross-reactive antibody response to a novel influenza A (H1N1) virus after vaccination with seasonal influenza vaccine.. MMWR Morb Mortal Wkly Rep.

[10] Hancock K, Veguilla V, Lu X, Zhong W, Butler EN (2009). Cross-reactive antibody responses to the 2009 pandemic H1N1 influenza virus.. N Engl J Med.

[11] (2009). Update: drug susceptibility of swine-origin influenza A (H1N1) viruses, April 2009.. MMWR Morb Mortal Wkly Rep.

[12] Hauge SH, Dudman S, Borgen K, Lackenby A, Hungnes O (2009). Oseltamivir-resistant influenza viruses A (H1N1), Norway, 2007–08.. Emerg Infect Dis.

[13] Dharan NJ, Gubareva LV, Meyer JJ, Okomo-Adhiambo M, McClinton RC (2009). Infections with oseltamivir-resistant influenza A(H1N1) virus in the United States.. JAMA.

[14] Rameix-Welti MA, Enouf V, Cuvelier F, Jeannin P, van der Werf S (2008). Enzymatic properties of the neuraminidase of seasonal H1N1 influenza viruses provide insights for the emergence of natural resistance to oseltamivir.. PLoS Pathog.

[15] Aoki FY, Boivin G, Roberts N (2007). Influenza virus susceptibility and resistance to oseltamivir.. Antivir Ther.

[16] de Jong MD, Tran TT, Truong HK, Vo MH, Smith GJ (2005). Oseltamivir resistance during treatment of influenza A (H5N1) infection.. N Engl J Med.

[17] (2009). Oseltamivir-resistant novel influenza A (H1N1) virus infection in two immunosuppressed patients - Seattle, Washington, 2009.. MMWR Morb Mortal Wkly Rep.

[18] (2009). Oseltamivir-resistant 2009 pandemic influenza A (H1N1) virus infection in two summer campers receiving prophylaxis–North Carolina, 2009.. MMWR Morb Mortal Wkly Rep.

[19] World Health Organization (WHO) Pandemic (H1N1) 2009-update 69.. http://www.who.int/csr/don/2009_10_09/en/index.html.

[20] Chen H, Cheung CL, Tai H, Zhao P, Chan JF (2009). Oseltamivir-resistant influenza A pandemic (H1N1) 2009 virus, Hong Kong, China.. Emerg Infect Dis.

[21] Baz M, Abed Y, Papenburg J, Bouhy X, Hamelin ME (2009). Emergence of oseltamivir-resistant pandemic H1N1 virus during prophylaxis.. N Engl J Med.

[22] Weinstock DM, Gubareva LV, Zuccotti G (2003). Prolonged shedding of multidrug-resistant influenza A virus in an immunocompromised patient.. N Engl J Med.

[23] Whitley RJ, Hayden FG, Reisinger KS, Young N, Dutkowski R (2001). Oral oseltamivir treatment of influenza in children.. Pediatr Infect Dis J.

[24] Moscona A (2005). Neuraminidase inhibitors for influenza.. N Engl J Med.

[25] Bantia S, Arnold CS, Parker CD, Upshaw R, Chand P (2006). Anti-influenza virus activity of peramivir in mice with single intramuscular injection.. Antiviral Res.

[26] Birnkrant D, Cox E (2009). The emergency use authorization of peramivir for treatment of 2009 H1N1 influenza.. N Engl J Med.

[27] Abed Y, Nehme B, Baz M, Boivin G (2008). Activity of the neuraminidase inhibitor A-315675 against oseltamivir-resistant influenza neuraminidases of N1 and N2 subtypes.. Antiviral Res.

[28] Herlocher ML, Truscon R, Elias S, Yen HL, Roberts NA (2004). Influenza viruses resistant to the antiviral drug oseltamivir: transmission studies in ferrets.. J Infect Dis.

[29] Ives JA, Carr JA, Mendel DB, Tai CY, Lambkin R (2002). The H274Y mutation in the influenza A/H1N1 neuraminidase active site following oseltamivir phosphate treatment leave virus severely compromised both in vitro and in vivo.. Antiviral Res.

[30] Baz M, Abed Y, Simon P, Hamelin ME, Boivin G (2010). Impact of the neuraminidase mutation H274Y conferring resistance to oseltamivir on the replicative capacity and virulence of old and recent human influenza A/H1N1 viruses.. J Infect Dis.

[31] Maines TR, Lu XH, Erb SM, Edwards L, Guarner J (2005). Avian influenza (H5N1) viruses isolated from humans in Asia in 2004 exhibit increased virulence in mammals.. J Virol.

[32] Tumpey TM, Maines TR, Van Hoeven N, Glaser L, Solorzano A (2007). A two-amino acid change in the hemagglutinin of the 1918 influenza virus abolishes transmission.. Science.

[33] Svitek N, Rudd PA, Obojes K, Pillet S, von Messling V (2008). Severe seasonal influenza in ferrets correlates with reduced interferon and increased IL-6 induction.. Virology.

[34] Fritz RS, Hayden FG, Calfee DP, Cass LM, Peng AW (1999). Nasal cytokine and chemokine responses in experimental influenza A virus infection: results of a placebo-controlled trial of intravenous zanamivir treatment.. J Infect Dis.

[35] Hayden FG, Fritz R, Lobo MC, Alvord W, Strober W (1998). Local and systemic cytokine responses during experimental human influenza A virus infection. Relation to symptom formation and host defense.. J Clin Invest.

[36] Skoner DP, Gentile DA, Patel A, Doyle WJ (1999). Evidence for cytokine mediation of disease expression in adults experimentally infected with influenza A virus.. J Infect Dis.

[37] Steel J, Staeheli P, Mubareka S, Garcia-Sastre A, Palese P (2010). Transmission of pandemic H1N1 influenza virus and impact of prior exposure to seasonal strains or interferon treatment.. J Virol.

[38] Kobinger GP, Meunier I, Patel A, Pillet S, Gren J (2010). Assessment of the efficacy of commercially available and candidate vaccines against a pandemic H1N1 2009 virus.. J Infect Dis.

[39] World Health Organization (WHO) (2010). Update on oseltamivir-resistant pandemic A (H1N1) 2009 influenza virus : January 2010.. Weekly Epidemiological Report.

[40] Baz M, Abed Y, Boivin G (2007). Characterization of drug-resistant recombinant influenza A/H1N1 viruses selected in vitro with peramivir and zanamivir.. Antivir Res.

[41] Liu Q, Wang S, Ma G, Pu J, Forbes NE (2009). Improved and simplified recombineering approach for influenza virus reverse genetics.. J Mol Genet Med.

[42] Abed Y, Baz M, Boivin G (2006). Impact of neuraminidase mutations conferring influenza resistance to neuraminidase inhibitors in the N1 and N2 genetic backgrounds.. Antivir Ther.

[43] Hoffmann E, Stech J, Guan Y, Webster RG, Perez DR (2001). Universal primer set for the full-length amplification of all influenza A viruses.. Arch Virol.

[44] Hatakeyama S, Sakai-Tagawa Y, Kiso M, Goto H, Kawakami C (2005). Enhanced expression of an alpha2,6-linked sialic acid on MDCK cells improves isolation of human influenza viruses and evaluation of their sensitivity to a neuraminidase inhibitor.. J Clin Microbiol.

[45] Reed LJ, Munch H (1938). A simple method of estimating fifty per cent endpoint.. Am J Hyg.

[46] Sergerie Y, Boivin G, Gosselin D, Rivest S (2007). Delayed but not early glucocorticoid treatment protects the host during experimental herpes simplex virus encephalitis in mice.. J Infect Dis.

[47] Hamelin ME, Couture C, Sackett MK, Boivin G (2007). Enhanced lung disease and Th2 response following human metapneumovirus infection in mice immunized with the inactivated virus.. J Gen Virol.

[48] Svitek N, von Messling V (2007). Early cytokine mRNA expression profiles predict Morbillivirus disease outcome in ferrets.. Virology.

