# Supplementary figures and images for: Oseltamivir-Resistant Pandemic A/H1N1 Virus Is as Virulent as Its Wild-Type Counterpart in Mice and Ferrets
Source: PLoS Pathog. 2010 Jul 22;6(7):e1001015. doi: 10.1371/journal.ppat.1001015 (PMC2908621; doi:10.1371/journal.ppat.1001015)

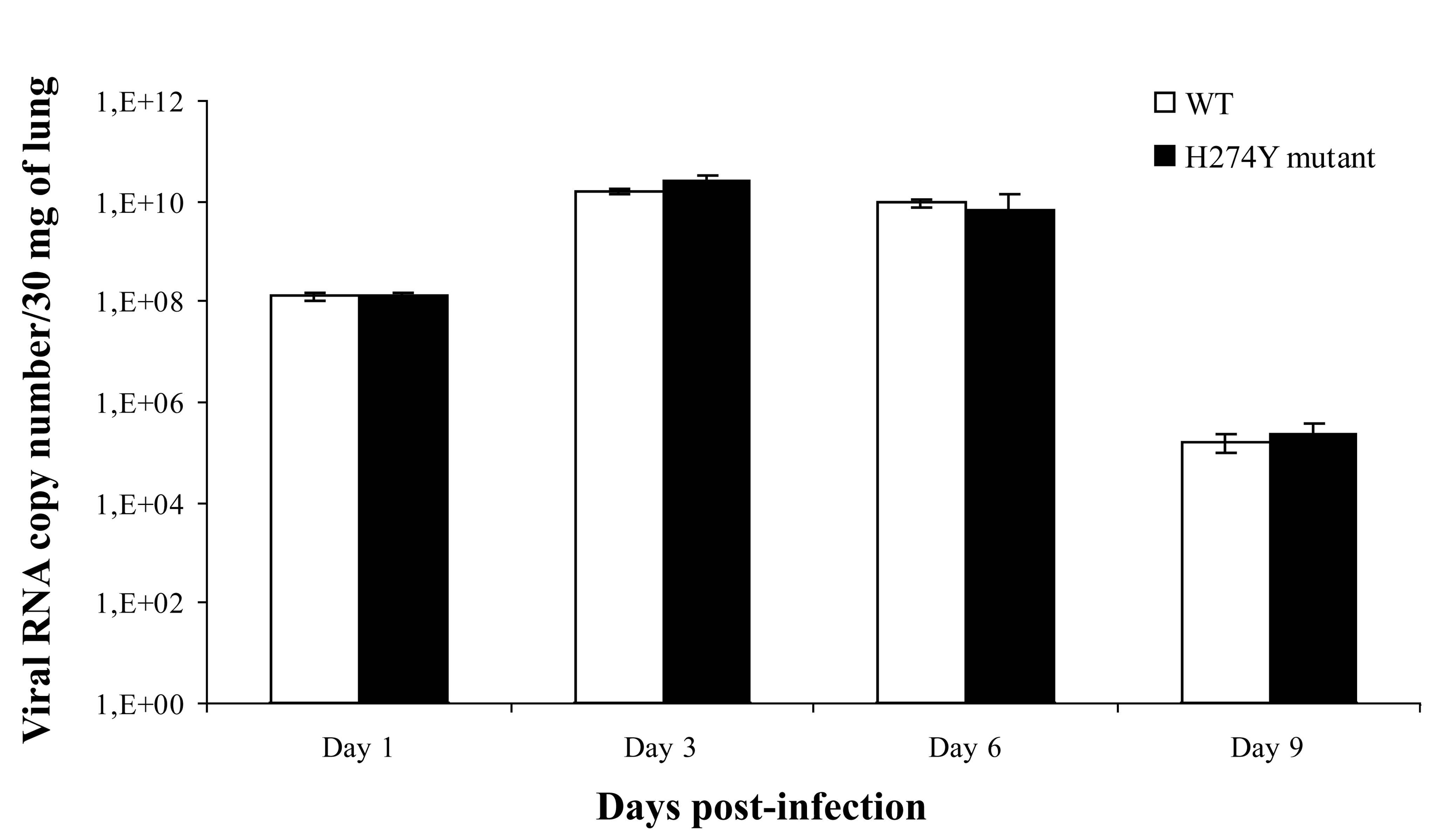

Supplement: Figure S1 — Lung viral titers of mice infected with wild-type (WT) or H274Y mutant isolates of pH1N1. (0.23 MB TIF) [file ppat.1001015.s001.tif]

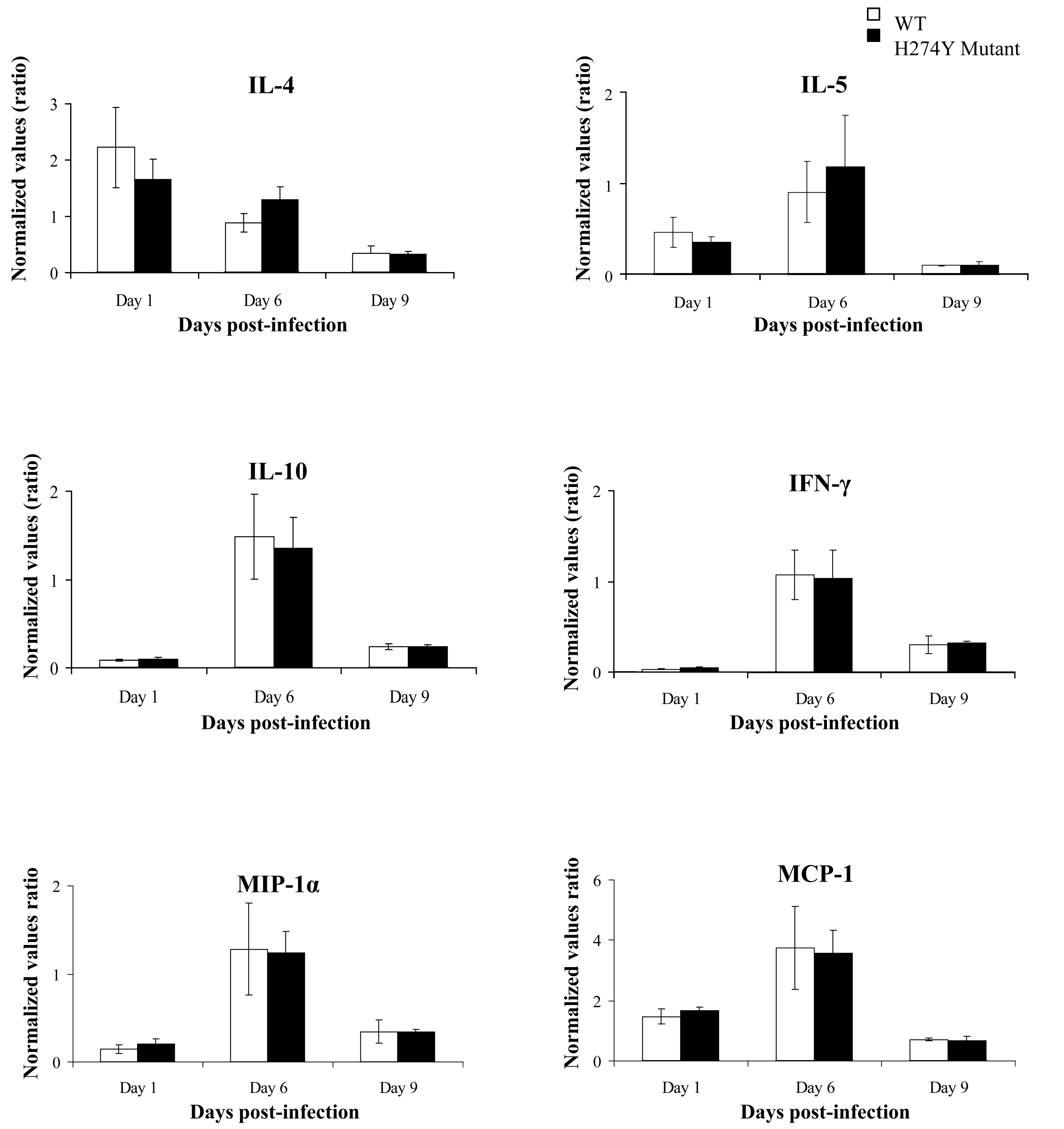

Supplement: Figure S2 — Lung cytokines/chemokines expression in mice infected with wild-type (WT) or H274Y mutant isolates of pH1N1. (0.41 MB TIF) [file ppat.1001015.s002.tif]

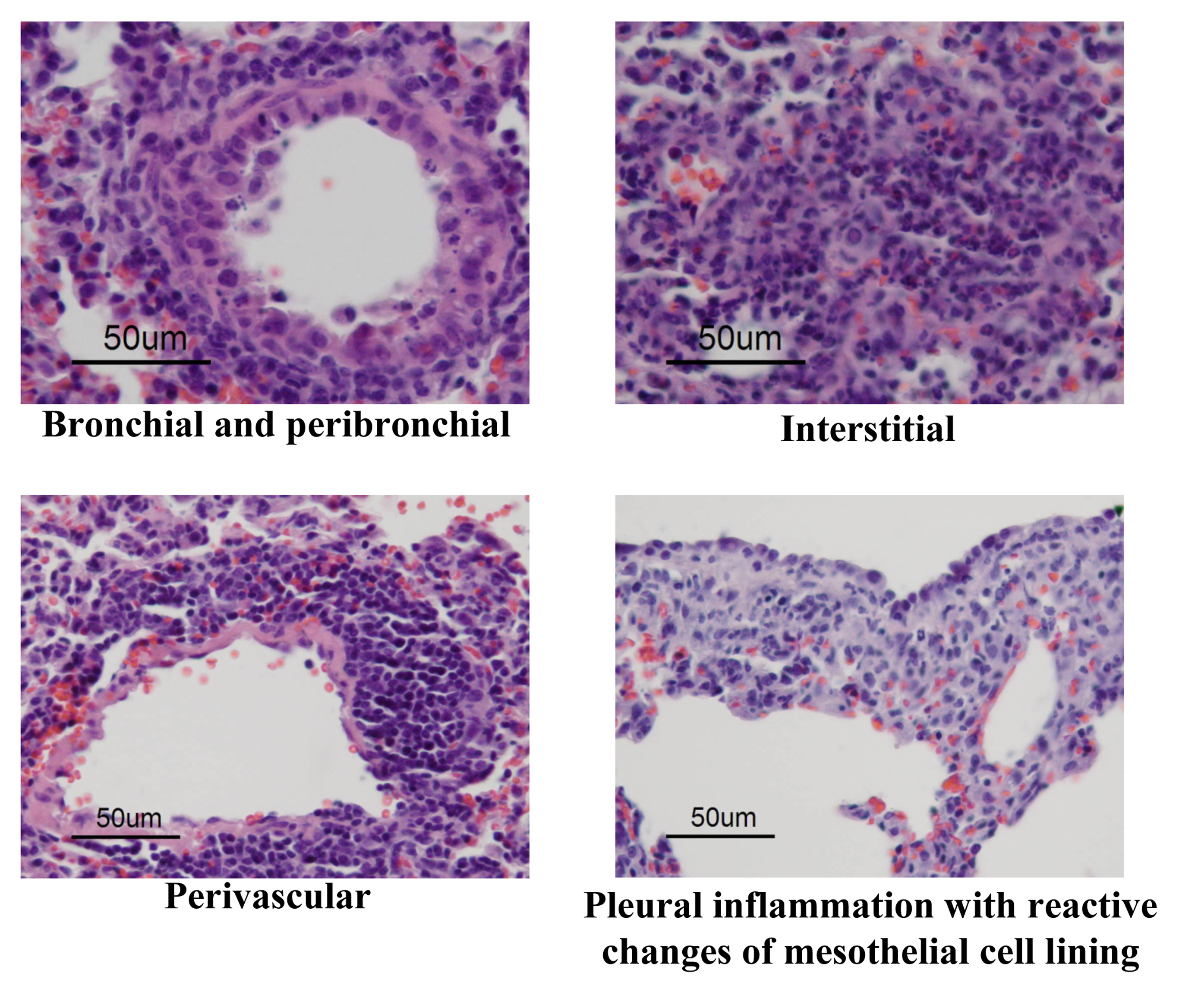

Supplement: Figure S3 — Lung histopathology of mice infected with wild-type (WT) or H274Y mutant isolates of pH1N1. (5.84 MB TIF) [file ppat.1001015.s003.tif]

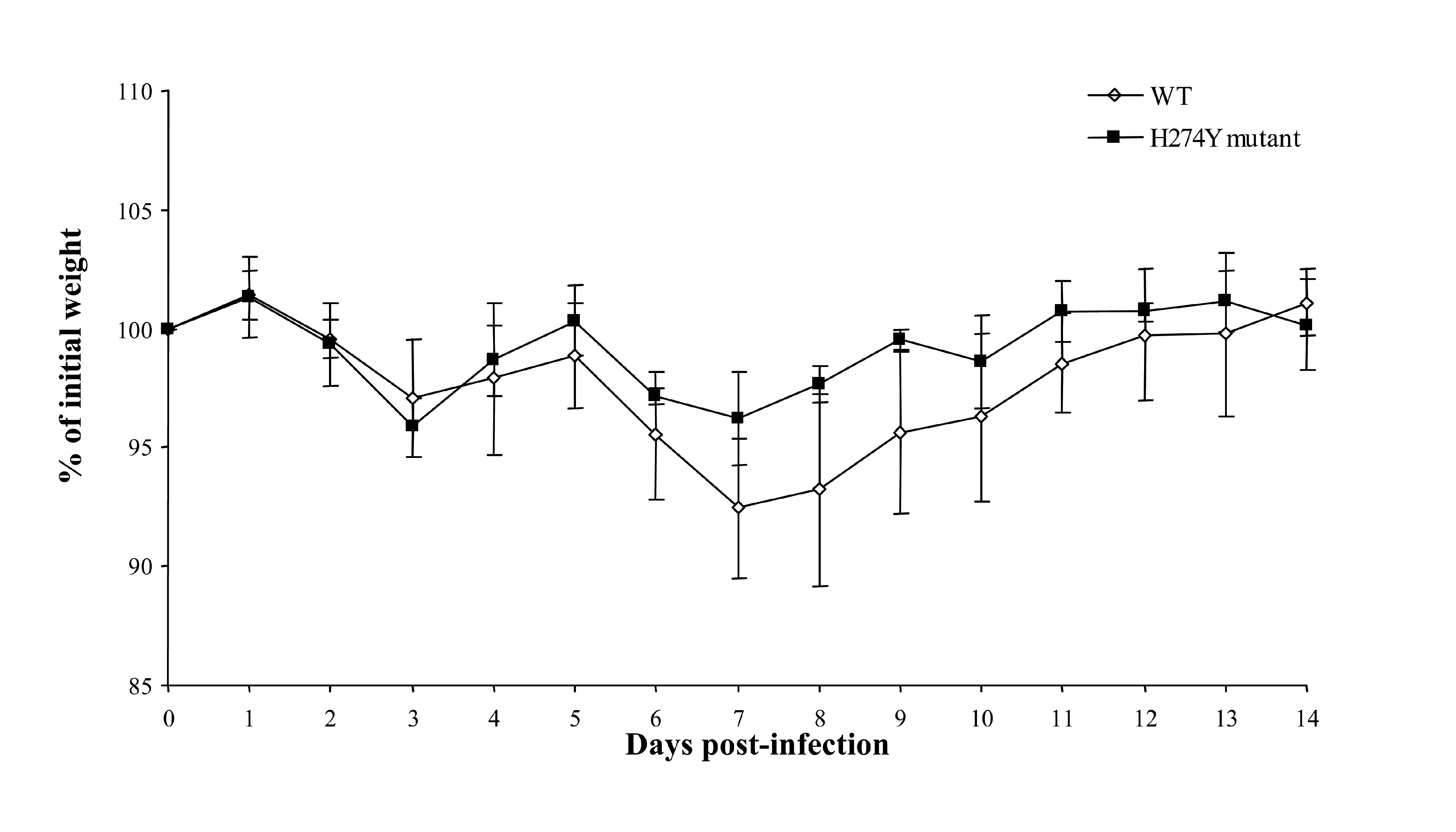

Supplement: Figure S4 — Weight loss of ferrets infected with wild-type (WT) or H274Y mutant isolates of pH1N1. (0.15 MB TIF) [file ppat.1001015.s004.tif]
