# Supplementary material for: Oseltamivir-Resistant Pandemic A/H1N1 Virus Is as Virulent as Its Wild-Type Counterpart in Mice and Ferrets
Source: PLoS Pathog. 2010 Jul 22;6(7):e1001015. doi: 10.1371/journal.ppat.1001015 (PMC2908621; doi:10.1371/journal.ppat.1001015)
Supplement: Table S1 — Hemagglutination inhibition titers for pH1N1 (A/California/7/09) and seasonal A/H1N1 (A/Brisbane/59/07) in ferrets infected with wild-type and H274Y mutant pH1N1 viruses. (0.04 MB DOC) [file ppat.1001015.s005.doc]

**Supplementary Table 1.** Hemagglutination inhibition titers for pH1N1 (A/California/7/09) and seasonal A/H1N1 (A/Brisbane/59/07) in ferrets infected with wild-type and H274Y mutant pH1N1 viruses.

|  | Pre-Infection Reciprocal HI Titers (Day 0) | | Post-Infection Reciprocal HI Titers (Day 14) | |
| --- | --- | --- | --- | --- |
| Ferret Number^1^ | Anti-A/California/7/09 | Anti-A/Brisbane/59/07 | Anti-A/California/7/09 | Anti-A/Brisbane/59/07 |
| 1 | <20 | 320 | 15,360 | 640 |
| 2 | <20 | 640 | 5,120 | 640 |
| 3 | <20 | 320 | 2,560 | 640 |
| 4 | <20 | 320 | 5,120 | 640 |
| 5 | <20 | 320 | 1,280 | 640 |
| 6 | <20 | 640 | 3,840 | 1,280 |
| 7 | <20 | 640 | 5,120 | 1,280 |
| 8 | <20 | 640 | 2,560 | 640 |
| 9 | <20 | 640 | 5,120 | 640 |
| 10 | <20 | 320 | 1,920 | 640 |

^1^Ferrets 1 through 5 were infected with wild-type pH1N1 whereas ferrets 6 through 10 were infected with H274Y mutant pH1N1 viruses.
